# Preliminary experience in the management of tracheobronchial foreign bodies in Lagos, Nigeria

**DOI:** 10.11604/pamj.2013.15.31.2710

**Published:** 2013-05-25

**Authors:** Bode Falase, Michael Sanusi, Adetinuwe Majekodunmi, Ifeoluwa Ajose, David Oke

**Affiliations:** 1 Cardiothoracic Division, Department of Surgery, Lagos State University College of Medicine, Lagos State University Teaching Hospital, Ikeja, Lagos, Nigeria; 2 Department of Anaesthesia, Lagos State University College of Medicine, Lagos State University Teaching Hospital, Ikeja, Lagos, Nigeria; 3 Department of Medicine, Lagos State University College of Medicine, Lagos State University Teaching Hospital, Ikeja, Lagos, Nigeria

**Keywords:** Bronchoscopy, Tracheobronchial Foreign Bodies, Lagos, Nigeria

## Abstract

Aspiration of tracheobronchial foreign bodies commonly affects young children, is potentially life threatening and requires early intervention for extraction. Access to facilities and skill manpower for bronchoscopic extraction is however limited in Nigeria. The aim of this study is to describe the experience in our institution with bronchoscopic removal of tracheobronchial foreign bodies and highlight the challenges encountered. This is a retrospective study of all patients referred to the Lagos State University Teaching Hospital with a diagnosis of tracheobronchial foreign body within the period of February 2008 and February 2013. Data extracted from the medical records were age, sex, time interval between aspiration and presentation, location of tracheobronchial foreign body, bronchoscopic technique, complications and outcome. A total of 24 patients were referred and confirmed at bronchoscopy to have tracheobronchial foreign bodies. Mean age was 6.6 + 5 years. Male to female ratio was 1:1. Delayed presentation was common with 22 patients (91.7%) presenting more than 24 hours after aspiration. Aspirated material was inorganic in 17 patients (70.8%) and organic in 7 patients (29.2%). Location of tracheobronchial foreign bodies was right main bronchus in 16 patients (66.7%), left main bronchus in 6 patients (25%) and the trachea in 2 patients (8.3%). Challenges to speedy and safe removal of the foreign bodies were delayed presentation and a limited range of bronchoscopic equipment early in the series which caused prolonged procedures and increased complications. Two mortalities occurred early in the series; one from airway obstruction and the other from respiratory failure caused by tracheobronchial oedema. Extraction of tracheobronchial foreign bodies was faster, more complete and safer later in the series due to a wider range of bronchoscopy equipment which included both flexible and rigid videobronchoscopy with the use of optical forceps. This preliminary experience suggests that an adequate armamentarium of bronchoscopy equipment is required to increase the chances of complete extraction, speed up the procedure and reduce the risk of complications of Tracheobronchial Foreign Bodies in our environment. Delayed presentation increases the difficulty of the procedure so earlier referral of these patients would help reduce the risk involved in their management.

## Introduction

Aspirated foreign bodies continue to present challenges to practitioners worldwide as well as in Nigeria. The major issues involve the accurate diagnosis and speedy and safe removal of the foreign bodies. Accurate diagnosis may be delayed as often the initial choking episode is not witnessed and the delayed residual symptoms may be minimal [1, 2]. The symptoms and signs produced depend upon the nature size, location and time since lodgment of the foreign body in the tracheobronchial tree [3]. A large foreign body occluding the upper airway may lead to sudden death whereas a small foreign body lodged in the tracheobronchial tree may cause less severe symptoms [4, 5]. Early diagnosis and treatment are imperative to prevent mortality as well as to prevent the complications of recurrent acute respiratory distress, chronic and recurrent pneumonia and pulmonary abscess.

Rigid bronchoscopy remains the gold standard for the removal of foreign bodies from the tracheobronchial tree under direct vision [6, 7]. The advent of ventilating bronchoscopes and improvement in the illumination and visualization provided by Hopkins telescope guided optical forceps and the advances in anaesthesia have reduced the mortality and greatly facilitated the task of the endoscopist by allowing simultaneous visualization and manipulation of the foreign bodies. Flexible bronchoscopy compliments rigid bronchoscopy and makes removal of foreign bodies even safer and more complete [8–10]. With modern bronchoscopy equipment, thoracotomy with bronchotomy and segmental resection of the lung as part of the management of bronchial foreign bodies has been largely relegated to the past [11, 12].

A different picture is however seen in practice in Nigeria and has been well described by several authors. Reported series have shown delayed presentation [13, 14], difficulty with confirmation of diagnosis [1], exclusive use of rigid bronchoscopy for foreign body extraction [1, 13, 15, 16], incomplete extractions [15, 16], the use of thoracotomy and bronchotomy [15] and mortalities from loss of airway secondary to bronchial oedema [15, 16]. The management of tracheobronchial foreign bodies is therefore seen to be very challenging in Nigeria.

The aim of this study is to describe our preliminary experience with bronchoscopic removal of tracheobronchial foreign bodies (TFB) and highlight our experience with addressing the unique challenges encountered in our environment.

## Methods

Following clinical and radiological assessment, arterial blood gases and serum electrolytes are obtained prior to transfer of the patient to the operating theatre. Patient monitoring in theatre includes heart rate and rhythm, pulse oximetry, non-invasive blood pressure and end tidal carbon dioxide for the duration of the procedure. Following intravenous induction of anaesthesia and skeletal muscle paralysis, patients are either endotracheally intubated and the airway secured in readiness for flexible bronchoscopy, or intubated primarily using the appropriate sized rigid bronchoscope. Anaesthesia was maintained using intravenous anaesthetic agents as large leaks during manipulations made inhalational anaesthesia extremely unreliable. Care was taken to ensure that ventilation was adequate and oxygen saturations were maintained above 95% throughout the procedure.

The bronchoscopic equipment used was all provided by Karl Storz (Tuttlingen, Germany). This includes a Medipack (combined camera control unit with LCD monitor), a flexible videobronchoscope (with flexible forceps), a paediatric fiberbronchoscope (with flexible forceps), rigid adult bronchoscope (with appropriate sized zero degree telescope and optical forceps), 2 rigid paediatric bronchoscopes (size 3.5 and size 6) with appropriate sized optical forceps and zero degree telescope. A telescope camera head adaptor ensured that videobronchoscopy was available for both rigid and flexible bronchoscopy.

Following successful extraction, patients are commenced on dexamethasone if the procedure was prolonged to reduce the risk of airway oedema. Patients are monitored on the ward for 24 hours. A repeat chest radiograph is done to assess the lung fields as well as document successful foreign body extraction prior to discharge home.

This is a retrospective study of all patients that were referred to the Cardiothoracic Division of the Lagos State University Teaching Hospital between February 2008 and February 2013 with a diagnosis of suspected TFB aspiration. Permission was obtained from our institutional ethics committee for the use of existing patient data extracted from the medical records. The data extracted included age, sex, time interval between aspiration and presentation, nature of foreign body, location of foreign body in the tracheobronchial tree, anaesthesia techniques, bronchoscopy techniques and outcome of bronchoscopy. Microsoft excel was used for data analysis. Summary data is presented as numbers, mean ± standard deviation and percentages as appropriate.

## Results

Twenty four patients were seen with TFBs in the study period. TFBs were confirmed at bronchoscopy in all patients and successfully extracted in 22 patients (91.7%). The male to female ratio was 1:1. The mean age was 6.6 ± 5 years and 75% of the patients were between the ages of 2-10 years (Table 1). The time interval between aspiration and presentation was very variable. Only 2 patients (8.3%) presented within 24 hours of aspiration and the remaining 22 patients (91.7%) presented at various time intervals beyond 24 hours (Table 2). The location of the TFB was the right main bronchus in 16 patients (66.7%), left main bronchus in 6 patients (25%) and the trachea in 2 patients (8.3%). The bronchoscopic technique used for extraction was Flexible in the first 5 patients (20.8%), Rigid in the next 2 patients (8.3%) and a combination of Flexible and Rigid Bronchoscopy in the most recent 17 patients (70.8%). The nature of the foreign body was inorganic in 17 patients (70.8%) and organic in 7 patients (29.2%). The exact nature of the foreign body in the different cases is as shown in Table 3.

**Table 1:** Age distribution of patients

Age (years)	No. of cases	Percentage (%)
**0-1**	2	8.3
**2-5**	8	25
**6-10**	12	50
**11-16**	1	4.2
**>16**	1	4.2

**Table 2:** Distribution of Time Intervals between aspiration and presentation

Time Interval	No. of cases	Percentage (%)
**< 1 day**	3 (12.5)	12.5
**2 – 7 days**	10 (41.7)	41.7
**8 – 30 days**	6 (25)	25
**>30 days**	5 (20.8)	20.8

**Table 3:** Nature of Tracheobronchial Foreign Body (TFB)

Inorganic		Organic	
Nature of TFB	No. of cases	Nature of TFB	No. of cases
Drawing Pin	4	Peanuts	4
Plastic Pen Cap	4	Fish Bone	1
Eraser	2	Breadfruit Seed	1
Nail	1	Crab	1
Metal Pen Clasp	1		
Watch Battery	1		
Ear Ring	1		
Metal Nut	1		
Metal Pen Cap	1		
Silver Foil Paper	1		
**TOTAL**	17	TOTAL	7

**Figure 1:**
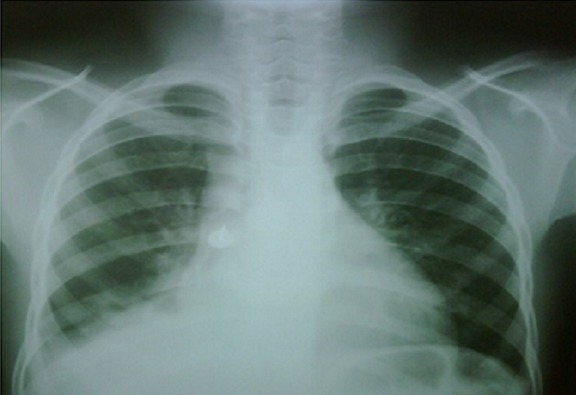
Chest Radiogram of drawing pin in the right main bronchus

**Figure 2:**
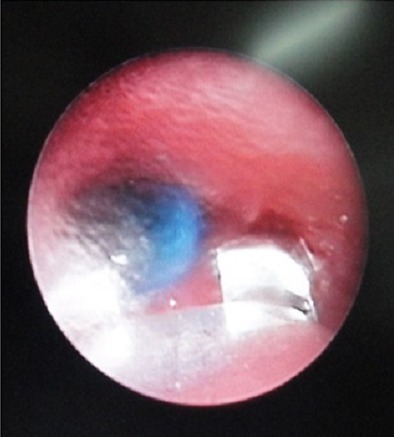
Optical forceps being used to remove plastic pen cap in the left main bronchus

Two mortalities were seen in this series (8.3%). Both occurred early in the series. The first was in the 2nd patient of the series in whom 7 peanuts were successfully extracted with flexible bronchoscopy but the patient succumbed to a respiratory arrest soon after extubation. The second mortality was seen in the 7th patient where a metallic nut was readily accessible with the rigid bronchoscope but difficulties during extraction stemmed from its asymmetrical shape and the limited range (at the time) of grasping instruments. The metal nut apparently migrated downwards causing acute airway obstruction with a sudden inability to ventilate. The patient was in the Intensive Care Unit awaiting tracheotomy to remove the foreign body. The 4^th^ patient in the series had a very late presentation (4 years) and a drawing pin was seen to be occluding the right main bronchus (Figure 1). Only the sharp point of the pin was visible and although the point could be grasped with the flexible alligator forceps it could not be dislodged from the surrounding tissues. Rigid equipment had not yet been acquired so the patient was referred to another teaching hospital where the foreign body was successfully extracted Figure 2. The 11th patient in the series was found to have a nail across the trachea, just above the carina. Adequate access to grasp the nail could not be obtained with either flexible or rigid bronchoscopy so a tracheotomy was performed while ventilation was maintained through an endotracheal tube. The nail was then successfully removed by rigid bronchoscopy through the tracheotomy using alligator optical forceps. The 13th patient in the series had a metal pen clasp in the right main bronchus which had become embedded in the surrounding tissue. The right main bronchus was too narrow to admit a larger bronchoscope to facilitate extraction. A Fogarty catheter was passed beyond the foreign body which was dislodged and brought up more proximally to the carina where it was removed using the larger bronchoscope.

## Discussion

Previous larger Nigerian series have shown that children are the age group mainly at risk for foreign body aspiration with mean age ranging between 1.7 and 4.8 years. There was also a propensity to mainly involve male children with most series showing 60-70% male predilection [1, 3, 4, 13, 14, 15, 16]. This is due to a combination of their natural curiosity, placing of various objects in the mouth and the small calibre of the airway which increases the risk of impaction [13, 14, 17]. The average age in our series was older at 6.6 ± 5 years and there was an equal male to female ratio.

The location of TFB in this series with the majority (66.7%) occurring in the right main bronchus is similar to the findings of previous Nigerian authors [1, 3, 13, 14, 15, 16]. This is thought to be as a result of the right main bronchus arising at a less acute angle from the trachea than the left main bronchus. The majority of aspirations in this series were inorganic (70.8%) with a preponderance of metallic objects. This is in contrast to previous Nigerian series where the majority of aspirations of TFBs have been shown to be organic. This may reflect a difference in the environment of the child at risk as Lagos is an urban centre whereas in most of the earlier Nigerian series the patients seen were largely from rural areas. Most patients in this study were of school age with increased access to inorganic materials. Many of the inorganic materials were objects that could be found in the school environment (Table 3). This is similar to the finding by Adegboye et al from Ibadan where most of the inorganic foreign body aspirations were seen in school children aged 6-10 years [13]. Another possibility is that organic materials are known to cause a severe type of chemical bronchitis and materials like peanuts and beans swell causing obstruction of the distal bronchi. Patients with inorganic TFB show less respiratory compromise and are therefore more likely to present as referrals to a tertiary centre [17]. As most patients in this series had a delayed presentation, those with organic TFB may have succumbed to a higher mortality in the community. It remains to be seen as more referrals accrue to our institution whether the proportion of inorganic to organic foreign bodies may change.

It is of concern that over a 5 year period only 24 patients presented to our institution with TFB giving an annual average of about 5 cases. This is lower than previous reports from Nigeria, where the yearly incidence was 7-10 cases [1, 13, 15, 16]. It would therefore appear that the number of cases seen in our institution may not represent a true picture of the actual magnitude of the problem in our environment as our institution serves a large urban population where children have ready access to small inorganic and organic objects. More awareness about the availability of resources for extraction in out hospitals needs to be raised as it is likely that many patients are dying of respiratory failure from TFB aspiration without being referred for extraction. This has been suggested by Adegboye et al in Ibadan [13] as 6 patients were brought in dead from TFB aspiration.

Other specific challenges to the management of TFB unique to our environment are the non-availability of a full range of bronchoscopy equipment for extraction of TFB in most centres, difficulty with confirmation of diagnosis, difficulty in performing full bronchoscopic evaluation for presence of TFB and delayed presentations which increase the difficulty of extraction. These are discussed below.

Full range of equipment: Previous Nigerian authors have reported experience of extraction of TFB exclusively limited to the use of rigid bronchoscopy. Videobronchoscopy does not appear to be in widespread use and neither is the use of optical forceps [1, 3, 13, 14, 15, 16]. In the series by Orji et al TFB was proven in 83% of the patients [1]. Ezeanuolue et al found no TFB in 13.3% of cases and 2 patients (6.6%) died of pulmonary oedema, likely from prolonged procedures [16]. Adeyemo et al had successful extraction of TFB in 73.9% of their cases. Three cases (6.5%) required tracheotomy and 9 cases (19.6%) required bronchotomy due to failure to extract TFB by rigid bronchoscopy. There were 3 mortalities (6.4%) from laryngospasm and complications of incomplete removal of vegetable seeds [15]. With the narrow calibre of the airway in the age group most prone to foreign body aspiration it has been shown that a full range of sizes of both flexible and rigid bronchoscopy equipment may be required for successful extraction [5, 6, 7, 8, 9, 10, 12]. Bronchoscopic options were limited to just flexible in our early experience which contributed to one of the mortalities and a failed extraction. The most recent 17 extractions have been successful as a full complement of flexible and bronchoscopic equipment has been available. This includes accessories like the dormia basket which was unavailable to extract a metal nut early in the series, contributing to the patient's mortality. A Fogarty catheter was used as an adjunct in another procedure which could otherwise have required a bronchotomy to extract the TFB. The use of optical forceps allows for visualization and manipulation within the narrow paediatric airway and has been a major contributor to the current high rate of successful extractions. An adequate range of equipment may therefore improve the ability to confirm the diagnosis of foreign body, increase the rate of successful extraction and reduce the complication of loss of airway seen from mucosal oedema secondary to prolonged manipulations in the tracheobronchial tree of children with small calibre airways. The need for open surgical intervention is also reduced. Bronchoscopic extraction of foreign bodies should however be performed with surgical backup as surgical intervention may occasionally be necessary for the extraction procedure as seen in the patient who required a tracheotomy to achieve foreign body extraction. It is advisable to proceed to surgical extraction if bronchoscopic extraction fails [12, 17].

Efforts to retrieve the foreign body should be well coordinated and require close cooperation between the surgical and anaesthetic teams. The team needs to recognize when efforts to retrieve the foreign body are causing excessive mucosal irritation and may lead to a compromised airway [9, 10, 13, 14, 17]. Airway compromise may have combined with migration of the foreign body in 7th patient in the series with the resultant mortality. Possible mucosal inflammation with the risk of airway compromise has influenced our recent use of dexamethasone perioperatively.

**Completeness of evaluation and confirmation of diagnosis:**Flexible bronchoscopy to localize the foreign body prior to rigid bronchoscopy has been described [8, 10, 12]. This may be performed under general anaesthesia passing the flexible bronchoscope through the endotracheal tube as we practice, or without endotracheal intubation under light sedation. Our choice of flexible bronchoscopy through an endotracheal tube has been influenced by the late presentation in most of our patients and the need to rapidly secure and maintain the airway.

**Delayed presentation:** Early referral of patients following foreign body aspiration should be encouraged. Delayed presentations are common in our environment [18]. Though Adegboye et al reported that 73% of patients in Ibadan presented within 2 hours of aspiration, 61.4% of the patients were under the age of 3 years, so these may have been mainly witnessed aspirations, increasing the speed of diagnosis [13]. Onotai et al reported that 68.75% of their patients presented more than 24 hours after aspiration, and the average age in that series was 3.8 years [4]. This delayed presentation is similar to our experience where 91.7% of patients presented more than 24 hours after aspiration. Delayed presentation often reflects a low index of suspicion leading to delay in referrals from private clinics or traditional healers. Delayed presentation is associated with a 2.5 times higher rate of serious acute complications than when the TFB is diagnosed early [2]. Parental education and public awareness would help in reducing the incidence and mortality [19].

## Conclusion

This preliminary experience suggests that an adequate armamentarium of bronchoscopic equipment is required to increase the chances of complete extraction, speed up the procedure and reduce the risk of complications of bronchoscopic extraction of TFB. Delayed presentation also increases the difficulty of the procedure so earlier referral of these patients would help reduce the risk involved in their management.

## References

[1] Orji FT, Akpeh JO (2011). Tracheobronchial foreign body aspiration in children: how reliable are clinical and radiological signs in the diagnosis?. Clin Otolaryngol.

[2] Wang K, Harnden A, Thomson A (2010). Easily missed? Foreign Body inhalation in children. Clinical Otolaryngology.

[3] Onotai LO, Ebong ME (2011). The pattern of foreign body impactions in the tracheobronchial tree in the University of Port Harcourt Teaching Hospital. Port Harcout Med J.

[4] Onotai LO, Ibekwe MU, George IO (2012). Impacted foreign bodies in the larynx of Nigerian children. J Med Med Sci.

[5] Lima JA (1989). Laryngeal foreign bodies in children: a persistent life threatening problem. The Laryngoscope.

[6] Korlacki W, Koreka K, Dzielicki J (2011). Foreign body aspiration in children: diagnostic and therapeutic role of bronchoscopy. Pediatr Surg Int.

[7] Surka AE, Chin R, Comforti J (2006). Bronchoscopic Myths and Legends; Airway Foreign Bodies. Clinical Myths and Evidence-based Medicine.

[8] Cutrone C, Pedruzzi B, Tava G, Emanuelli E, Barion U, Fischetto D (2011). The complimentary role of diagnostic and therapeutic endoscopy in foreign body aspiration in children. Int J Pediatr Otorhinolaryngol.

[9] Dikensoy O, Usalan C, Filiz A (2002). Foreign body aspiration; Clinical utility of flexible bronchoscopy. Postgrad Med J.

[10] Swanson KL, Prakash UB, Midthun DE, Edell ES, Utz JP, McDougall JC (2002). Flexible bronchoscopic management of airway foreign bodies in children. Chest.

[11] Kaur K, Sonkhya N, Bapna AS (2002). Foreign bodies in the Tracheobronchial Tree: a prospective study of fifty cases. Ind J Otolaryng and Head and Neck Surgery.

[12] Rodrigues JA (2012). Bronchoscopic techniques for removal of foreign bodies in children's airways. Paediatr Pulmonol.

[13] Toff WD, Camm AJ, Skehan JD (2005). Single-chamber versus dual chamber pacing for high-grade atrioventricular block. N Engl J Med.

[14] Bhatia PL (1991). Problems in the management of aspirated foreign bodies. West African Journal of Medicine.

[15] Adeyemo AO, Bankole MA (1986). Foreign bodies in the tracheobronchial tree: management and complications. J Natl Med Assos.

[16] Ezeanuolue BC, Izuora KLA, Ezike HA (2003). Tracheobronchial foreign bodies in Nigerian children: Clinical profile and a technique of administering anaesthesia during rigid bronchoscopic removal. J College Med.

[17] De Sousa ST (2009). Foreign body aspiration in children and adolescents; experience of a Brazilian referral center. J Bras Pneumol.

[18] Okafor BC (1995). Foreign bodies in the larynx, clinical features and a plea for early referral. Niger Med J.

[19] Iversen RH, Klug TE (2012). Need for more clear parental recommendations regarding foreign body aspiration in children. Dan Med J.

